# Epidemiology of Invasive Pneumococcal Disease in Older People in Spain (2007–2009): Implications for Future Vaccination Strategies

**DOI:** 10.1371/journal.pone.0043619

**Published:** 2012-08-22

**Authors:** Carmen Ardanuy, José María Marimón, Laura Calatayud, Montserrat Giménez, Marta Alonso, Immaculada Grau, Román Pallarés, Emilio Pérez-Trallero, Josefina Liñares

**Affiliations:** 1 Department of Microbiology, Hospital Universitari de Bellvitge-University of Barcelona-IDIBELL, Barcelona, Spain; 2 CIBER de Enfermedades Respiratorias (CIBERES) ISCIII, Madrid, Spain; 3 Department of Microbiology, Hospital Univeristario Donostia, Donostia, Spain; 4 Department of Microbiology, Hospital Germans Trias i Pujol, Barcelona, Spain; 5 Department of Infectious Diseases, Hospital Universitari de Bellvitge-University of Barcelona-IDIBELL, Barcelona, Spain; Rockefeller University, United States of America

## Abstract

**Background:**

Recently, the 13-valent pneumococcal conjugate vaccine (PCV13) has been recommended for adults. We analyzed the epidemiology of invasive pneumococcal disease (IPD) in older adults in Spain before PCV13 introduction.

**Methodology/Principal Findings:**

IPD episodes, defined as clinical findings together with an invasive pneumococcal isolate, were prospectively collected from patients aged over 65 years in three hospitals in Spain from 2007 to 2009. A total of 335 IPD episodes were collected. Pneumonia was the main clinical syndrome, while chronic obstructive pulmonary disease, diabetes mellitus and cancer were the main underlying diseases. Pneumococcal isolates were serotyped and the molecular typing was performed by PFGE/MLST. PCV13 serotypes accounted for 59.3% of isolates, the most prevalent being serotypes 19A (15.1%), 3 (9.6%), 7F (7.5%), 14 (6.9%) and 1 (5.4%). The most frequent non-PCV13 serotypes were serotypes 16F (4.5%), 22F (3.6%), 24F (3.3%) and 6C (2.1%). The most common genotypes were CC230 (8.5%, serotypes 19A and 24F), CC156 (8.2%, serotypes 9V and 14), ST191 (7.9%, serotype 7F), CC260 (6.6%, serotype 3), ST306 (5.2%, serotype 1), CC30 (4.6%, serotype 16F) and ST433 (3.6%, serotype 22F). Comparing the 335 IPD isolates to 174 invasive pneumococci collected at the same hospitals in 1999–2000, PCV7 serotypes decreased (45.4% vs 18.4%,p<0.001), non-PCV7 serotypes included in PCV13 increased (26.4% vs 41.0%,p = 0.001) and two non-PCV13 serotypes increased (serotype 6C 0% vs 2.1%, p = 0.05; serotype 24F 0.6% vs 3.3%, p = 0.04,).

**Conclusion:**

In our older adult population two serotypes (19A and 3) included in PCV13 accounted for about a quarter of IPD episodes in people ≥65 years. Non-PCV13 emerging serotypes should be carefully monitored in future surveillance studies.

## Introduction

*Streptococcus pneumoniae* is a major cause of morbidity and mortality worldwide, it being responsible for a wide variety of invasive diseases such as bacteremic pneumonia, septicemia and meningitis [1]. Children under 2 years of age and adults over 65 are the two most important groups with a higher risk of invasive pneumococcal disease (IPD). The nasopharynx of children, especially those attending day-care centers, is usually colonized by pneumococci, which may spread to adults.

Pneumococci are divided into at least 93 different serotypes, and some capsular polysaccharides have been used to develop vaccines. Since the late 1970s adult vaccination has been based on pneumococcal polysaccharide vaccines (PPV). Currently, PPV23 is recommended for people over 65 and adults at risk of IPD [2]. Since PPV23 has low effectiveness against children under 5 (especially those under 18 months), conjugate pneumococcal vaccines have been developed, with the 7-valent pneumococcal conjugate vaccine (PCV7) being licensed in 2000 in the US, and since 2001 in most European countries.

Despite the implementation of PPV23 in older adults no significant changes in the incidence of IPD, serotype distribution and penicillin resistance were observed during the 1990s in Spain [3], [4]. The most important change in the 1990s was the increase in macrolide resistance rates, which was probably associated with the introduction of long-acting macrolides and the spread of mobile elements carrying macrolide resistance determinants [3]–[5]. However, after introduction of PCV7 for children, IPD caused by PCV7 serotypes, as well as antibiotic resistance rates, decreased in children in the US and in many other countries [6]–[10]. This decrease was also observed in adults, especially those aged over 65, due to herd protection [8], [10]–[13]. The surveillance of pneumococcal serotypes/clones in these countries identified the evasion of vaccine immunity as being due to the increase of non-vaccine serotypes and clones, as well as to the emergence of new clones produced by capsular switching events [9]–[11], [14], [15].

Two new pneumococcal conjugate vaccines are currently available for child vaccination and they include 10 (PCV10) and 13 (PCV13) pneumococcal serotypes. The indirect effect of child vaccination observed in adults, especially those older than 65, has increased interest worldwide in using these new conjugate vaccines in adult vaccination. In 2011, PCV13 was approved for adult vaccination schedules in the US and Europe.

In this study we aimed to analyze the serotype and genotype composition of invasive pneumococci isolated from older adults before the introduction of PCV13 in three Spanish hospitals. In addition, we also compared the current serotype and genotype distribution to that studied previously (1999–2000) in order to identify the emerging serotypes/clones that are not included in PCV13.

**Table 1:** Demographics, source of isolates and clinical characteristics of IPD episodes.

	1999–2000		2007–2009	
	n = 174		n = 335	
age (mean±SD)		75.9±7.0		76.4±8.1
Male sex	100	57.5%	194	57.9%
Source of strains				
Blood	150	86.2%	292	87.2%
CSF	9	5.2%	19	5.7%
Pleural fluid	12	6.9%	22	6.6%
Ascitic fluid	3	1.7%	2	0.6%
Clinical syndromes				
Pneumonia	139	79.9%	269	80.3%
Meningitis	12	6.9%	28	8.4%
Peritonitis	8	4.6%	5	1.5%
Bacteremia without focus	9	5.2%	20	6.0%
Septic arthritis	1	0.6%	1	0.3%
Otomastoiditis	1	0.6%	3	0.9%
Biliar	3	1.7%	3	0.9%
Cellulitis	0	0%	2	0.6%
No data	1	0.6%	4	1.2%
Main underlying diseases				
COPD	40	23.0%	76	22.7%
Cirrhosis	15	8.6%	13	3.9%
Diabetes Mellitus	37	21.3%	80	23.9%
Malignance	39	22.4%	103	30.7%
Renal failure	7	4.0%	30	9.2%

## Results

### A Total of 335 IPD Episodes were Studied in Older Adults from 2007 to 2009

Table 1 shows the demographic characteristics, source of strains, clinical syndromes and the main underlying diseases (2007–2009 vs. 1999–2000). The most important source of strains was blood cultures, with pneumonia being the main clinical syndrome. The main underlying diseases were chronic obstructive pulmonary disease (COPD), diabetes mellitus, cancer and chronic renal failure.

Table 2 shows the antibiotic susceptibility to 8 antimicrobials of 335 pneumococci collected from invasive disease. Using oral breakpoints (classical), more than a quarter of the isolates showed penicillin non-susceptibility and 7.5% showed cefotaxime non-susceptibility. However, it should be noted that when using current non-meningeal CLSI breakpoints for parenteral penicillin and cefotaxime 98.5% of isolates were fully susceptible, which supports the use of beta-lactams in the treatment of non-meningeal infections. Although rates of erythromycin, tetracycline and cotrimoxazol resistance are lower than those reported previously in Spain, the non-susceptibility rates of these antibiotics remains above 20% [3], [4]. Chloramphenicol and ciprofloxacin showed good in vitro activity.

Among 332 serotyped pneumococci collected in 2007–2009 the five most frequent serotypes were: 19A (n = 50, 15.1%), 3 (n = 32, 9.6%), 7F (n = 25, 7.5%), 14 (n = 23 6.9%) and 1 (n = 18, 5.4%). The proportion of IPD isolates included in PCV7 was 18.4%, with the proportions being 33.7% in PCV10, 59.3% in PCV13 and 73.8% in PP23V. The most frequent non-PCV13 serotypes were serotypes 16F (4.5%), 22F (3.6%), 24F (3.3%) and 6C (2.1%).

Table 3 shows the STs associated with major serotypes. Overall, the most frequent genotypes among IPD isolates from the 2007–2009 period were CC230 (n = 26, 8.5%), CC156 (n = 25, 8.2%), ST191 (n = 24, 7.9%), CC260 (n = 20, 6.6%), ST306 (n = 16, 5.2%), CC30 (n = 14, 4.6%) and ST433 (n = 11, 3.6%).

During this period (2007–2009) pneumonia was the most frequent cause of IPD (n = 269 episodes). Among these cases the most frequent serotypes were 19A (n = 43, 16.2%), 3 (n = 25, 9.4%), 7F (n = 23, 8.6%) and 14 (n = 20, 7.5%). PCV10 serotypes accounted for 34.2% of pneumonia isolates and PCV13 for 60.9%. In this period, 21 pneumococci were collected from pleural fluid and the most frequent serotypes were 1 (n = 3), 3 (n = 3), 19A (n = 2) and 14 (n = 2).

Among 28 isolates causing meningitis in the 2007–2009 period the most frequent serotypes were 3 (n = 4), 6C (n = 3), 19A (n = 3), 5 (n = 2), 8 (n = 2), 24F (n = 2), 23A (n = 2) and 23F (n = 2). Using current CLSI breakpoints for meningitis, no cefotaxime resistance was detected among these 28 meningeal isolates (MIC range <0.03–0.5 µg/ml), although 17.8% of them were penicillin resistant (range 0.12–0.5 µg/ml). The three IPD episodes related to otomastoiditis were caused by serotypes 3, 14 and 22F.

Four serotypes (19A, n = 22; 14, n = 19; 24F, n = 10; and 9V, n = 6) accounted for 66.3% of penicillin non-susceptible isolates (MIC ≥0.12 µg/ml), the most frequent CC being CC230 (n = 26, 31.7%) and CC156 (n = 25, 30.5%). The main serotypes among cefotaxime non-susceptible isolates were 14 (n = 11), 19A (n = 8) and 9V (n = 5), whereas a single clonal complex CC156 (n = 17) accounted for 68% of these isolates.

Serotypes 19A (n = 21), 24F (n = 10) and 16F (n = 8) accounted for 49.5% of macrolide-resistant strains, which were related to CC230 (n = 19), CC30 (n = 7) and CC63 (n = 6) and accounted for 41.9% of strains. These macrolide-resistant serotypes and clones were associated with clindamycin and tetracycline resistance, probably due to the presence of transposons of the Tn916-family, which carry macrolide- and tetracycline-resistant genes [5].

**Table 2:** Antimicrobial susceptibility of 335 invasive pneumococci collected from adults ≥65 years old (2007–2009).

	MIC50	MIC90	%I	%R
Antibiotic	µg/ml			
Penicillin (meningeal)	≤0.03	1	–	25.7
Penicillin (non- meningeal)	≤0.03	1	1.5	0
Penicillin (classical)	≤0.03	1	20.0	5.7
Cefotaxime (meningeal)	≤0.06	0.5	6.0	1.5
Cefotaxime (no-meningeal)	≤0.06	0.5	1.5	0
Erythromycin	≤0.5	≥128	0	23.7
Clindamycin	≤0.25	≥128	0	19.9
Tetracycline	≤0.25	32	6.4	16.8
Chloramphenicol	≤2	≤2	–	6.4
Cotrimoxazol	≤0.5	≥2	3.1	26.3
Ciprofloxacin	1	1	–	2.1

Since pneumococci were collected from three hospitals located in two Spanish regions the serotype and genotype differences were analyzed. Serotype 19A was more prevalent in Basque Country than in Catalonia [21/90 (23.3%) vs 29/242; (12%) p = 0.01] whereas serotype 24F was only detected among pneumococci from Catalonia [11/242 (4.5%) vs 0/90 (0%); p = 0.038]. These differences were associated with genotype differences: two serotype 19A genotypes were only detected in the Basque Country (CC199 and ST193); and the prevalence of CC230 (which includes serotype 24F and 19A) was more frequent in Catalonia [23/220 (10.5%) vs 3/85 (3.5%), p = 0.052].

### Recent Changes in Serotypes and Genotypes

**Figure 1:**
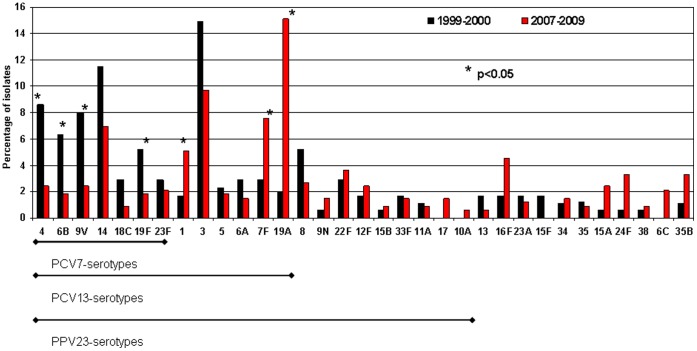
Serotype distribution of invasive pneumococci collected from adults over 65 by period. The serotype distribution in the two periods analyzed 1999–2000 (pre-PCV7, n = 174 isolates) and 2007–2009 (pre-PCV13, n = 332 isolates) is showed. Statistically significant changes are indicated with an asterisk. Overall, PCV7 serotypes decreased from 45.4% (1999–2000) to 18.4% (2007–2009, p<0.001). Non-PCV7 serotypes included in PCV13 increased from 26.4% to 41.0% (p = 0.001). Serotype 6A is not included in PPV23. In 2007–2009 period, the proportion of IPD isolates included in PCV7 was 18.4%, with the proportions being 59.3% in PCV13 and 73.8% in PP23V.

Figure 1 shows the serotype distribution in the two periods analyzed: 1999–2000 (pre-PCV7) and 2007–2009 (pre-PCV13). In the 2007–2009 period there was an overall decrease in the frequency of PCV7 serotypes (4, 6B, 9V, 14, 18C, 19F and 23F), from 45.4% (n = 79) to 18.4% (n = 61) (p<0.001). However, there was an overall increase in the frequency of non-PCV7 serotypes included in PCV13 (1, 3, 5, 7F, 6A and 19A), from 26.4% (n = 46) in 1999–2000 to 41.0% (n = 136) in 2007–2009 (p = 0.001). Serotype 19A increased from 2.0% in 1999–2000 to 15.1% in 2007–2009, a rise of 65.9% (95% CI, 16.9% to 203.8%). Two serotypes not included in PCV13 increased in the 2007–2009 period: serotypes 6C (from 0% to 2.1%, p = 0.16) and 24F (from 0.6% to 3.3%, p = 0.066). No statistically significant differences in serotype distribution were observed between patients aged 65–74 years and those who were older (data not shown).

**Table 3:** Distribution of genotypes associated with predominant serotypes by time period.

	Period 1999–2000			Period 2007–2009		
Serotype	No.IPD	Studied	STs	No.IPD	Studied	STs
1	3	2	304 (n = 1), 306 (n = 1)	18	17	304 (n = 1); 306 (n = 16)
3	26	24	180 (n = 5); 260 (n = 15); 1220 (n = 3); 1253 (n = 1)	32	29	180 (n = 8); 260 (n = 15); 1220 (n = 4); 1253 (n = 2)
4	15	13	247(n = 13)	8	7	247 (n = 7)
6B	11	11	90 (n = 5); 315 (n = 3); 386 (n = 2); 490 (n = 1)	6	6	90 (n = 1); 138 (n = 1); 315 (n = 2); 1624 (n = 1); 386(n = 1)
6C	0	0	–	7	6	224 (n = 3); 4011 (n = 1); 4310 (n = 1); 4534 (n = 1)
7F	5	4	191 (n = 4)	25	25	191 (n = 24), 4815 (n = 1)
8	9	8	53 (n = 7); 3219 (1)	9	8	53 (n = 5); 4816 (n = 1); 1629 (n = 2)
9V	14	14	66 (n = 1); 156 (n = 12); 280 (n = 1)	8	8	62 (n = 1); 156 (n = 5); 4796 (n = 1); 378 (n = 1)
14	20	19	17 (n = 4); 67 (n = 2); 156 (n = 12); 1554 (n = 1)	23	22	9 (n = 3); 67 (n = 1); 156 (n = 18)
16F	3	3	30 (n = 2); 4022 (n = 1)	15	14	30 (n = 12); 4022 (n = 1); 570 (n = 1)
19A	4	3	202 (n = 1); 276 (n = 1); 1201 (n = 1)	50	49	63 (n = 1); 81 (n = 1); 156 (n = 1); 193 (n = 5); 199 (n = 7);202 (n = 1); 276 (n = 12); 320 (n = 3); 433 (n = 1); 645(n = 1); 994 (n = 2); 1201 (n = 10); 2013 (n = 4)
19F	9	8	63 (n = 3); 88 (n = 1); 157 (n = 2); 177 (n = 2)	6	6	63 (n = 1); 88 (n = 3); 89 (n = 1); 177 (n = 1)
22F	5	5	433 (n = 4); 1372 (n = 1)	12	11	433 (n = 10); 3241 (n = 1)
24F	1	1	72 (n = 1)	11	11	230 (n = 10); 72 (n = 1)
12F	3	3	218 (n = 2); 4004 (n = 1)	9	6	989 (n = 6)

**Figure 2:**
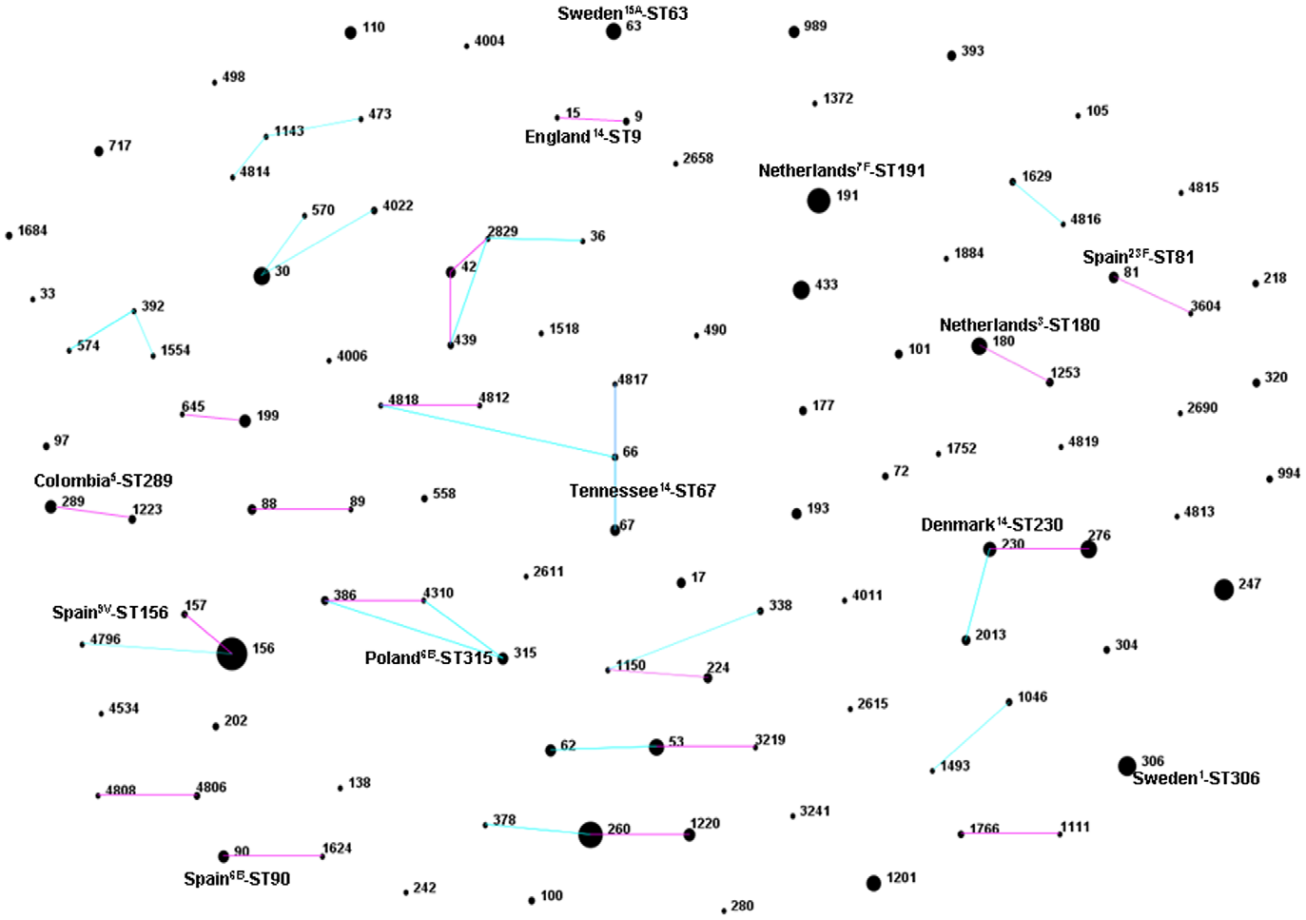
Population structure of invasive *S. pneumoniae* collected from older people in Spain. The overall eBURST analysis of MLST data for 463 invasive pneumococci showed 22 clonal groups and 46 singletons (blue lines: single locus variant; and pink lines: double locus variant). The most frequent genotypes were: CC156 (11.2%), CC260 (8.2%), ST191 (6.0%), CC230 (5.8%), ST247 (4.3%), CC53/62 (4.1%), CC30 (3.7%), ST306 (3.7%), CC180 (3.5%) and ST433 (3.2%).

Figure 2 shows the overall (including isolates of both periods) eBURST analysis of pneumococcal genotypes. A total of 22 clonal groups and 46 singletons were found. The most frequent genotypes among IPD episodes were: CC156 (n = 52, 11.2%), CC260 (n = 38, 8.2%), ST191 (n = 28, 6.0%), CC230 (n = 27, 5.8%), ST247 (n = 20, 4.3%), CC53/62 (n = 19, 4.1%), CC30 (n = 17, 3.7%), ST306 (n = 17, 3.7%), CC180 (n = 16, 3.5%) and ST433 (n = 15, 3.2%).

Table 3 shows the genotypes related to predominant serotypes by period. Due to the low genetic diversity of some serotypes the changes in the frequency of these serotypes was related to genotype changes. For instance, the increase in serotype 1 was associated with a significant increase in the ST306 clone (0.6% vs. 5.2%), while the increase in serotype 7F was related to the spread of the ST191 clone (2.5% vs. 7.9%). The decrease in serotype 4 paralleled the decrease in ST247 (8.2% vs. 2.3%). The high genetic diversity of other serotypes such as serotype 6C resulted in no genotype-related changes. Finally, the changes in clonal complexes (CC), which included isolates with different capsular types, reflected the serotype increase or decrease on a different scale. For instance, the multidrug-resistant CC230 increased markedly (0.6% vs. 8.5%) because this CC includes STs of serotypes 24F (ST230) and 19A (ST276 and ST2013), whose frequency increased in the second period. Another example was the decrease in CC156 (17.1% vs. 8.2%), which was related to the decrease in serotypes 9V and 14.

It is interesting to note that despite the high genetic diversity of serotype 19A, the alarming rise in this serotype was mainly reflected in the increase of the previously mentioned CC230 (ST276 and ST2013; 0.6% vs. 5.6%) and three STs: ST1201 (0.6% vs. 3.3%), ST199 (0% vs. 2.6%) and ST193 (0% vs. 1.6%).

## Discussion

Since the introduction of PCV7 for children important changes in IPD have occurred in many countries. People over 65 are at increased risk for developing IPD, mainly because of the ageing process and associated co-morbidities. For these reasons pneumococcal immunization has been recommended in this group of patients [2]. Prior to 2011 only the 23-valent pneumococcal polysaccharide vaccine was available for adult immunization, but recently the pediatric conjugate vaccine with an enhanced immunological response, PCV13, has been approved for adult vaccination in Europe and the US. Knowledge of the current serotype distribution of pneumococci causing IPD in adults over 65 would therefore be helpful in order to establish the current coverage of this new vaccine in this age group.

In this way, three European regions recently replaced PCV7 for PCV13 (Germany in December 2009, England and Wales in April 2010 and the Autonomous Region of Madrid in June 2010) in routine childhood vaccination. In these regions an early effect of reduction of IPD has been observed in children under 2 years (target population). Moreover a reduction of IPD due to serotypes 19A and 7F has been demonstrated in England and Wales. These preliminary data support the beneficial effect that could be achieved after PCV13 vaccination of adults. [16], [17], [18].

In Spain the PCV7 vaccine for children was introduced in 2001 but it was not subsidized by the government, with exception of the Autonomous Region of Madrid which started universal childhood vaccination in November 2006 (more than 95% of children vaccinated). Currently, in this Spanish region PCV7 serotypes accounted for less than 5% of pediatric IPD episodes [16]. However, in the rest of the country, the use of PCV7 did increase on a voluntary basis through the private sector, and childhood vaccination rates (excluding the Madrid Region) are estimated to be around 50–60% [4], [10], [11]. In agreement with other studies carried out in countries where PCV7 has been used, we observed a marked decrease in PCV7 serotypes among pneumococci causing IPD in older adults due to herd protection [8], [10]–[13]. Overall, a major benefit of decreasing PCV7 serotypes was the fall in antibiotic resistance. However, other non-PCV7 serotypes increased, mainly due to clonal expansion [10], [11], [14], [15], [19], [20]. For instance, the rise in serotypes 1 and 7F in the 2007–2009 period was associated with the clonal expansion of Sweden^1^-ST306 and Netherlands^7F^-ST191, respectively, as observed during the first decade of this century in most European countries [11], [19]–[22]. However, the most notorious was the dramatic increase in serotype 19A, as observed in many countries after PCV7 introduction, especially in children under 5 years of age [4], [6], [11], [12], [23]. In our study, serotype 19A is currently the first cause of invasive disease in older adults, as reported recently in France; furthermore, and as described previously, serotype 19A pneumococci were multiclonal [14], [19], [21]. The important role acquired by serotype 19A makes it necessary to include this serotype in the routine vaccination of older adults. Moreover, multidrug resistance, which is frequent in this serotype, is related to two clones: CC230 and CC320. CC320 is the major contributor to the increase in resistant serotype 19A pneumococci in the US and in Asia, but only 3 isolates from the present study belonged to this CC [14], [24], [25]. Here, as in most other studies from other European countries, the multidrug resistant strains are mainly associated with ST276 or ST2013, which are single locus and double locus variants of Denmark^14^-ST230 [18], [25]. In fact, CC230, which also includes isolates of serotype 24F with ST230, ranks first among invasive isolates in the present study.

In 2010, PCV13 replaced PCV7 for child vaccination, and PCV13 has recently been approved for adults. Given the results obtained after PCV7 introduction one would expect to see a progressive decrease in PCV13 serotypes among older adults over the next few years. In fact, in the UK when the universal PCV13 vaccination started in April 2010 a decrease in PCV13 serotypes (mainly 7F and 19A) has been observed in children and also in adults by herd protection [17]. In this scenario, however, it is important to know which serotypes could be prone to increase in the near future. Our results showed an increase in serotype 24F (non-PCV13) associated with the expansion of the ST230 clone, which was described in Italy as a cause of meningitis [26]. However, this serotype-genotype association (24F-ST230) was only detected in Catalonia. As regards serotype 6C, which emerged in the 2007–2009 period accounting for 2.1% of invasive isolates, the increase was multiclonal. Although serotype 6C is not included in the current PCV13, cross-protection between serotypes 6A and 6C has been reported [27]. Surveillance studies are therefore needed in order to determine the impact of PCV13 on serotype 6C trends. The increases observed in other non-PCV13 serotypes such as 22F, 12F, 16F and 35B may suggest that these serotypes could play an important role in adult IPD in the near future. In fact, serotypes 22F, 12F and 16F had low genetic diversity thus the observed increase was due to a clonal expansion (ST433, ST989, and CC30, respectively). Then, a surveillance of these serotypes and genotypes should be performed to know the impact of PCV13 introduction.

Regarding clinical manifestations we observed no important changes, with pneumonia being the most common cause of IPD in people over 65 and accounting for 80% of IPD episodes. Contrary to what has been observed in children in our country, we found no increase in empyema in these patients, probably because serotype 1 is less frequent in older adults than it is in children [22]. The burden of IPD in older adults is an increasing problem in many parts of the world, mainly due to the associated co-morbidities and the increased life expectancy. In our study we observed no significant changes in co-morbidities, with the exception of cancer, which probably reflects the increased rate of cancer diagnoses among the elderly.

The impact of child PCV7 vaccination on IPD in older adults (herd protection) that has been demonstrated in the present study, as well as by others, reinforces the relevance of adult vaccination with conjugate vaccines [8], [10]–[13]. In Europe, PCV10 and PCV13 are currently used for children, and the latter has recently been approved for adults over 50. Although we have not addressed this study to adults 50–65, 64.7% of IPD occurred in people over 50 in 2007–2009 period in the three hospitals of the present study were caused by serotypes included in PCV13. In the current scenario, and given the efficacy of PCV7, the preliminary data of PCV13 vaccination, and the coverage of PCV13 for adults over 65 (59.3%) or over 50 (64.7%), PCV13 could be a good vaccination option for the elderly in Spain. However, this study is focused on two Spanish regions and some regional differences have been observed, therefore it is necessary to have local data in order to establish the PCV13 coverage in each region. Finally, further studies will be needed in order to evaluate the effects of PCV13 introduction.

## Methods

### Ethical Statement

This study and publication of the results were approved by the “Comité Ètic d’Investigació Clínica del Hospital Universitari de Bellvitge” and written or oral informed consent was considered not necessary, because data were analyzed anonymously.

### Population and Invasive Disease Surveillance

We prospectively studied episodes of invasive pneumococcal disease (IPD) diagnosed in adults ≥65 years in three Spanish hospitals, two located in the region of Catalonia (Hospital Universitari de Bellvitge and Hospital Universitari Germans Trias i Pujol) and one in the Basque Country (Hospital Universitario Donostia). IPD was defined as the presence of clinical symptoms together with the growth of pneumococci in blood or other sterile fluid samples (e.g., CSF, pleural fluid or ascitic fluid).

PCV7 was licensed for child vaccination in Spain in June 2001, with PCV10 being licensed in April 2009 and PCV13 in June 2010. In order to identify changes in the serotype/genotype distribution and emerging serotypes/clones not included in the conjugate vaccines, two periods were compared: the current distribution (2007–2009) and the distribution before the introduction of conjugate vaccines (1999–2000). Data concerning serotype and genotype distributions for the 1999–2000 period have been partially published [10], [11].

### Bacterial Srains, Serotyping and Antimicrobial Susceptibility Tests

A total of 509 invasive pneumococci were analyzed from older adults. Of these, 335 were collected in the 2007–2009 period and 174 in the 1999–2000 period. Serotype was determined by the Quellung reaction and/or dot-blot assay or PCR in all 174 isolates from the 1999–2000 period and in 332 (99.1%) isolates from the 2007–2009 period [28], [29].

Antimicrobial susceptibility to penicillin, cefotaxime, erythromycin, clindamycin, chloramphenicol and tetracycline were tested by the microdilution method, following the procedures and criteria of the Clinical Laboratory Standards Institute [30], [31]. *S. pneumoniae* ATCC 6303 and *S. pneumoniae* ATCC 49619 were used as control strains.

### Molecular Typing

A total of 463 (91.0%) available isolates were studied by pulsed-field gel electrophoresis (PFGE) after Smal restriction [305 (91.1%) from the 2007–2009 period and 158 (90.8%) isolated in the 1999–2000 period] [32]. PFGE patterns were compared to representative international pneumococcal clones from the Pneumococcal Molecular Epidemiology Network (PMEN) [32].

Selected isolates were studied by multilocus sequence typing (MLST), as described previously [33]. These isolates were representative of major PFGE clusters (those accounting for more than 3 isolates). The allele number and sequence types (ST) were assigned by using the pneumococcal MLST website (http://www.mlst.net), which is hosted at Imperial College London and is funded by the Wellcome Trust. When an unusual association between serotype and sequence type was found, the serotype was confirmed by PCR, using previously described methodology (http://www.cdc.gov/ncidod/biotech/strep/pcr.htm), and MLST was repeated.

### Statistical Analysis

Statistical analyses were performed using SPSS for Windows, version 17.0 (SPSS). We used X^2^ or Fisher’s exact tests to compare proportions, as appropriate. A P value of <0.05 was considered statistically significant.

## References

[1] Musher DM (1992) Infections caused by *Streptococcus pneumoniae*: clinical spectrum, pathogenesis, immunity, and treatment. Clin Infect Dis 14: 801–807. 1576274 10.1093/clinids/14.4.801

[2] Centers for Disease Control and Prevention (CDC); Advisory Committee on Immunization Practices (2010) Updated recommendations for prevention of invasive pneumococcal disease among adults using the 23-valent pneumococcal polysaccharide vaccine (PPSV23). MMWR Morb Mortal Wkly Rep 59: 1102–6. 20814406

[3] Fenoll A, Granizo JJ, Aguilar L, Giménez MJ, Aragoneses-Fenoll L, et al (2009) Temporal trends of invasive *Streptococcus pneumoniae* serotypes and antimicrobial resistance patterns in Spain from 1979 to 2007. J Clin Microbiol 47: 1012–20. 19225097 10.1128/JCM.01454-08 PMC2668361

[4] Liñares J, Ardanuy C, Pallares R, Fenoll A (2010) Changes in antimicrobial resistance, serotypes and genotypes in *Streptococcus pneumoniae* over a 30-year period. Clin Microbiol Infect 16: 402–10. 20132251 10.1111/j.1469-0691.2010.03182.x

[5] Calatayud L, Ardanuy C, Tubau F, Rolo D, Grau I, et al (2010) Serotype and genotype replacement among macrolide-resistant invasive pneumococci in adults. Mechanisms of resistance and association with different transposons. J Clin Microbiol 48: 1310–6. 20147647 10.1128/JCM.01868-09 PMC2849543

[6] Hicks LA, Harrison LH, Flannery B, Hadler JL, Schaffner W, et al (2007) Incidence of pneumococcal disease due to non-pneumococcal conjugate vaccine (PCV7) serotypes in the United States during the era of widespread PCV7 vaccination, 1998–2004. J Infect Dis196: 1346–1354. 10.1086/521626 17922399

[7] Kyaw MH, Lynfield R, Schaffner W, Craig AS, Hadler J, et al (2006) Effect of introduction of the pneumococcal conjugate vaccine on drug-resistant *Streptococcus pneumoniae* . N Engl J Med 354: 1455–1463. 16598044 10.1056/NEJMoa051642

[8] Whitney CG, Farley MM, Hadler J, Harrison LH, Bennett NM, et al (2003) Decline in invasive pneumococcal disease after the introduction of protein-polysaccharide conjugate vaccine. N Engl J Med. 348: 1737–1746. 10.1056/NEJMoa022823 12724479

[9] Byington CL, Samore MH, Stoddard GJ, Barlow S, Daly J, et al (2005) Temporal trends of invasive disease due to *Streptococcus pneumoniae* among children in the intermountain west: emergence of nonvaccine serogroups. Clin Infect Dis 41: 21–29. 15937758 10.1086/430604

[10] Pérez-Trallero E, Marimon JM, Ercibengoa M, Vicente D, Pérez-Yarza EG (2009) Invasive *Streptococcus pneumoniae* infections in children and older adults in the north of Spain before and after the introduction of the heptavalent pneumococcal conjugate vaccine. Eur J Clin Microbiol Infect Dis 28: 731–738. 19153783 10.1007/s10096-008-0693-1

[11] Ardanuy C, Tubau F, Pallares R, Calatayud L, Domínguez MA, et al (2009) Epidemiology of invasive pneumococcal disease among adult patients in Barcelona before and after pediatric 7-valent pneumococcal conjugate vaccine introduction, 1997–2007. Clin Infect Dis 48: 57–64. 19035779 10.1086/594125

[12] Miller E, Andrews NJ, Waight PA, Slack MP, George RC (2011) Herd immunity and serotype replacement 4 years after seven-valent pneumococcal conjugate vaccination in England and Wales: an observational cohort study. Lancet Infect Dis 11: 760–768. 21621466 10.1016/S1473-3099(11)70090-1

[13] Lexau CA, Lynfield R, Danila R, Pilishvili T, Facklam R, et al (2005) Changing epidemiology of invasive pneumococcal disease among older adults in the era of pediatric pneumococcal conjugate vaccine. JAMA 294: 2043–2051. 16249418 10.1001/jama.294.16.2043

[14] Moore MR, Gertz RE Jr, Woodbury RL, Barkocy-Gallagher GA, Schaffner W, et al (2007) Population snapshot of emergent *Streptococcus pneumoniae* serotype 19A in the United States, 2005. J Infect Dis 197: 1016–1027. 10.1086/528996 18419539

[15] Brueggemann AB, Pai R, Crook DW, Beall B (2007) Vaccine escape recombinants emerge after pneumococcal vaccination in the United States. PLoS Pathog 3: 1628–1636. 10.1371/journal.ppat.0030168 PMC2077903 18020702

[16] Picazo J, Ruiz-Contreras J, Casado-Flores J, Giangaspro J, García-de Miguel MJ, et al.. (2012) First impact data of 13-valent pneumococcal conjugate vaccine (PCV13) on invasive pneumococcal disease in children in Madrid, 2010–2011 (Heracles study). 8th. International Symposium on Pneumococci and Pneumococcal Diseases. Poster number 189.

[17] Andrews N, Kaye P, Slack M, George R, Miller E (2012) Effectiveness of the 13 valent pneumococcal conjugate vaccine against IPD in England and Wales. 8th. International Symposium on Pneumococci and Pneumococcal Diseases. Poster number 148.

[18] M. van der Linden, M Imöhl (2012) Effects of immunization with higher valent pneumococcal conjugate vaccines in German children on numbers of reported IPD cases. 8th. International Symposium on Pneumococci and Pneumococcal Diseases. Poster No 199.

[19] Sá-Leão R, Pinto F, Aguiar S, Nunes S, Carriço JA, et al (2011) Analysis of invasiveness of pneumococcal serotypes and clones circulating in Portugal before widespread use of conjugate vaccines reveals heterogeneous behavior of clones expressing the same serotype. J Clin Microbiol 49: 1369–1375. 21270219 10.1128/JCM.01763-10 PMC3122870

[20] Beall B, McEllistrem MC, Gertz RE Jr, Wedel S, Boxrud DJ, et al (2006) Active Bacterial Core Surveillance Team. Pre- and postvaccination clonal compositions of invasive pneumococcal serotypes for isolates collected in the United States in 1999, 2001, and 2002. J Clin Microbiol 44: 999–1017. 16517889 10.1128/JCM.44.3.999-1017.2006 PMC1393141

[21] Grall N, Hurmic O, Al Nakib M, Longo M, Poyart C, et al (2011) Epidemiology of *Streptococcus pneumoniae* in France before introduction of the PCV-13 vaccine. Eur J Clin Microbiol Infect Dis 30: 1511–1519. 21499971 10.1007/s10096-011-1251-9

[22] Obando I, Muñoz-Almagro C, Arroyo LA, Tarrago D, Sanchez-Tatay D, et al (2008) Pediatric parapneumonic empyema, Spain. Emerg Infect Dis 14: 1390–1397. 18760005 10.3201/eid1409.071094 PMC2603109

[23] Pelton SI, Huot H, Finkelstein JA, Bishop CJ, Hsu KK, et al (2007) Emergence of 19A as virulent and multidrug resistant pneumococcus in Massachusetts following universal immunization of infants with pneumococcal conjugate vaccine. Pediatr Infect Dis J 26: 468–472. 17529860 10.1097/INF.0b013e31803df9ca

[24] Choi EH, Kim SH, Eun BW, Kim SJ, Kim SH, et al (2008) *Streptococcus pneumoniae* serotype 19A in children, South Korea. Emerg Infect Dis 14: 275–281. 18258121 10.3201/eid1402.070807 PMC2600206

[25] Ardanuy C, Rolo D, Fenoll A, Tarrago D, Calatayud L, et al (2009) Emergence of a multidrug-resistant clone (ST320) among invasive serotype 19A pneumococci in Spain. J Antimicrob Chemother 64: 507–510. 19535383 10.1093/jac/dkp210

[26] Pantosti A, Gherardi G, Conte M, Faella F, Dicuonzo G, et al (2002) A novel, multiple drug-resistant, serotype 24F strain of *Streptococcus pneumoniae* that caused meningitis in patients in Naples, Italy. Clin Infect Dis 35: 205–208. 12087529 10.1086/341250

[27] Cooper D, Yu X, Sidhu M, Nahm MH, Fernsten P, et al (2011) The 13-valent pneumococcal conjugate vaccine (PCV13) elicits cross-functional opsonophagocytic killing responses in humans to *Streptococcus pneumoniae* serotypes 6C and 7A. Vaccine 29: 7207–7211. 21689707 10.1016/j.vaccine.2011.06.056 PMC3170457

[28] Fenoll A, Jado I, Vicioso D, Casal J (1997) Dot blot assay for the serotyping of pneumococci. J Clin Microbiol 35: 764–766. 9041430 10.1128/jcm.35.3.764-766.1997 PMC229668

[29] Marimon JM, Monasterio A, Ercibengoa M, Pascual J, Prieto I, et al (2010) Antibody microarray typing, a novel technique for *Streptococcus pneumoniae* serotyping. J Microbiol Methods 80: 274–80. 20093147 10.1016/j.mimet.2010.01.011

[30] Clinical and Laboratory Standard Institute (2009) Methods for dilution antimicrobial susceptibility test for bacteria that growth aerobically; 8^th^ ed. Approved standard. CLSI/NCCLS document M7-A8. Wayne, Pa: Clinical and Laboratory Standards Institute. Vol 29.

[31] Clinical Laboratory Standard Institute (2011) Performance Standards for Antimicrobial Susceptibility Testing; Twenty-first Informational Supplement. CLSI/NCCLS document M100-S21. Wayne, Pa: Clinical and Laboratory Standards Institute. Vol 31.

[32] McGee L, McDougal L, Zhou J, Spratt BG, Tenover FC, et al (2001) Nomenclature of major antimicrobial-resistant clones of *Streptococcus pneumoniae* defined by the pneumococcal molecular epidemiology network. J Clin Microbiol 39: 2565–2571. 11427569 10.1128/JCM.39.7.2565-2571.2001 PMC88185

[33] Enright MC, Spratt BG (1998) A multilocus sequence typing scheme for *Streptococcus pneumoniae*: identification of clones associated with serious invasive disease. Microbiology 144: 3049–3060. 9846740 10.1099/00221287-144-11-3049

